# A Sensitive Electrochemical Sensor Based on Calix[n]arene-Modified PGE for the Determination of Tofacitinib in Human Urine Samples and Pharmaceutical Dosage Forms

**DOI:** 10.1021/acsomega.6c01716

**Published:** 2026-06-05

**Authors:** Tugba Sardohan Koseoglu, Aykut Yalabik, Hasan Koseoglu

**Affiliations:** † 565593 Isparta University of Applied Sciences, Faculty of Technology, Department of Biomedical Engineering, Isparta 32100, Turkey; ‡ Isparta Provincial Health Directorate, Directorate of Public Hospitals Services, Isparta 32100, Turkey; § 52994 Suleyman Demirel University, Faculty of Engineering, Department of Environmental Engineering, Isparta 32260, Turkey

## Abstract

Tofacitinib is a potent Janus kinase inhibitor widely used in the treatment of various autoimmune diseases, including rheumatoid arthritis and psoriasis. Monitoring its concentration is vital for therapeutic efficacy and safety. In this study, a novel, rapid, and highly sensitive electrochemical sensing platform was developed for tofacitinib determination using pencil graphite electrodes (PGEs) modified with polypyrrole (PPy) and calix­[n]­arene derivatives. The modified electrodes were prepared via cyclic voltammetry (CV) by incorporating three different macrocycles: calix[4]­arene, calix[6]­arene, and calix[8]­arene. Surface characterization was performed using Scanning Electron Microscopy (SEM) and SEM-EDS. Under optimized conditions (Britton–Robinson buffer at pH 4.0, 5 polymerization cycles, and 0.005 M modifier concentration), tofacitinib was quantified using differential pulse voltammetry (DPV). The linear calibration ranges were established as 0.10–0.80 ppm for calix[4]­arene and calix[6]­arene, and 0.10–0.60 ppm for calix[8]­arene. The limits of detection (LOD) were found to be 0.0275, 0.0212, and 0.0184 ppm for calix[4], [6], and [8] derivatives, respectively, demonstrating a clear correlation between macrocyclic cavity size and analytical sensitivity. The proposed sensor exhibited excellent selectivity in the presence of common interferents like ascorbic acid, caffeine, and glucose. Furthermore, the practical applicability of the method was validated in pharmaceutical formulations and human urine samples, yielding satisfactory recovery values between 98.0% and 101.5%. This study provides a cost-effective and disposable alternative to sophisticated chromatographic methods for routine clinical analysis.

## Graphical abstract

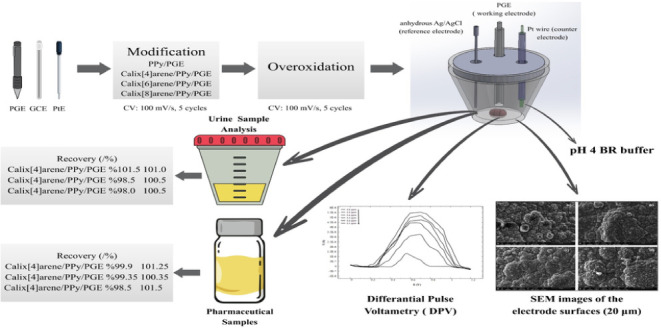

## 1 Introduction

Calixarenes are a prominent class of macrocyclic compounds extensively utilized as chemical sensors across various scientific disciplines, including medical diagnostics, environmental monitoring, and supramolecular chemistry. Due to their unique structural characteristics, namely, their cage-like, vase-shaped cavities and their ease of synthesis and functionalization compared to other macrocyclic compounds, calixarenes are widely favored in host–guest chemistry.^1,2^ Their ability to selectively encapsulate metal cations and organic molecules within their well-defined cavities has made them valuable receptor molecules in supramolecular synthesis and applications.

The term “calixarene” was first popularized in the 1970s through the pioneering work of C. David Gutsche, who demonstrated that calixarenes of varying ring sizes could be synthesized via the condensation of p-alkylphenols with formaldehyde.^3^ Structurally, calixarenes are composed of phenolic units linked via methylene bridges, forming a rigid, three-dimensional, cylindrical cavity. This cavity offers two distinct chemical environments: the exorim, which consists of the p-positions of the phenolic rings, and the endorim, where the phenolic hydroxyl groups reside.^4,5^

Tofacitinib citrate is a small molecule belonging to the Janus kinase (JAK) inhibitor class. Discovered and developed by Pfizer, it was approved by the U.S. Food and Drug Administration (FDA) in 2012 for the treatment of autoimmune diseases.^6,7^ Tofacitinib functions as a reversible and competitive inhibitor, targeting the ATP-binding site within the catalytic domain of JAK kinases.^8^ It is currently approved for clinical use in treating several autoimmune conditions, including rheumatoid arthritis, psoriasis, and, more recently, moderate to severe ulcerative colitis. Compared to biologic disease-modifying antirheumatic drugs (DMARDs) such as abatacept, adalimumab, and etanercept, tofacitinib has demonstrated superior efficacy and is frequently used as monotherapy.^9^ Despite its therapeutic benefits, the clinical use of tofacitinib citrate is associated with various side effects that necessitate careful patient monitoring. Common adverse effects include headaches, diarrhea, nasopharyngitis, and upper respiratory tract infections. More importantly, serious safety concerns have been reported, such as an increased risk of blood clots (deep vein thrombosis and pulmonary embolism), gastrointestinal perforations, and a heightened susceptibility to opportunistic infections like pneumonia or tuberculosis due to its immunosuppressive nature. Furthermore, long-term use has been linked to a potential increase in the risk of certain malignancies, including lymphoma. The severity of these side effects, coupled with the drug’s narrow therapeutic index, underlines the critical importance of developing sensitive and rapid analytical methods for monitoring tofacitinib levels in biological fluids to optimize treatment and minimize toxicity.^10^ Despite its clinical significance, the determination of tofacitinib in complex matrices has been predominantly limited to chromatographic techniques,^6,11–14^ which, while sensitive, often require expensive instrumentation, time-consuming sample pretreatment, and high consumption of organic solvents. The literature survey presents various analytical strategies for tofacitinib determination across different matrices. While high performance liquid chromatography (HPLC) and reversed phase high performance liquid chromatography (RP-HPLC) methods have been optimized for rat plasma,^13^ human serum,^14^ and pharmaceutical dosage forms,^6,11,12^ recent electrochemical studies have focused on glassy carbon electrode (GCE) and boron-doped diamond electrode (BDDE) surfaces for analysis in human serum.^15,16^ Our study extends this scope by utilizing a disposable and cost-effective pencil graphite electrode (PGE) platform for both pharmaceutical and urine samples, providing a practical alternative for routine clinical monitoring.

In this study, a novel electrochemical sensor system was developed for the voltammetric determination of tofacitinib citrate. The synergy between the high surface area of polypyrrole (PPy) and the molecular recognition capabilities of calixarenes was expected to enhance the preconcentration of tofacitinib onto the electrode surface via host–guest interactions. PPy, a conductive polymer known for its ability to form stable films on various electrode surfaces, was employed in the electrode modification process. Electrodes were fabricated using calix[4]­arene, calix­[6]­arene, and calix[8]­arene incorporated into PPy on PGEs via the cyclic voltammetry (CV) method. The electrochemical behavior of tofacitinib citrate at these modified electrodes (calix[4]­arene/PPy/PGE, calix[6]­arene/PPy/PGE, calix[8]­arene/PPy/PGE) was then investigated using differential pulse voltammetry (DPV).

The enhancement in voltammetric response at the modified electrode interface is attributed to the supramolecular host–guest recognition between calixarene cavities and tofacitinib molecules. The sensing mechanism is primarily governed by a combination of π-π stacking interactions between the aromatic rings of tofacitinib and the phenolic scaffold of the calixarene, as well as potential hydrogen bonding between the nitrogen-containing functional groups of the analyte and the phenolic hydroxyl groups at the calixarene’s endorim. Furthermore, the inclusion of the tofacitinib molecule within the macrocyclic cavity facilitates its preconcentration on the electrode surface. The superior sensitivity observed with calix[8]­arene, as evidenced by the lowest limits of detection (LOD) value, is attributed to its larger macrocyclic framework, which provides a more sterically compatible cavity for the tofacitinib molecule and a higher density of phenolic interaction sites, thereby enhancing the host–guest binding affinity. This synergistic effect, combined with the high conductivity and increased surface area provided by the PPy matrix, promotes faster electron transfer kinetics and significantly lowers the limit of detection compared to unmodified electrodes.

Although various analytical methods have been employed for tofacitinib citrate determination, electrochemical studies remain limited. Specifically, recent reports have described its voltammetric determination using GCE and BDDE,^15^ as well as a more complex sensing platform based on molecularly imprinted polymers (MIP).^16^ However, to the best of our knowledge, no study has yet explored the use of PGE modified with calix­[n]­arene derivatives for this purpose. This study introduces a novel and cost-effective approach by synergizing the high surface area and disposability of PGE with the unique host–guest recognition capabilities of calix­[n]­arenes. By doing so, this work fills a significant gap in the literature, providing a sensitive, reliable, and portable platform that offers a practical alternative to more expensive and time-consuming chromatographic or complex sensing techniques.

## 2 Materials and Methods

### 2.1 Chemicals and Reagents

Tofacitinib citrate (≥97.5%, Acros, Germany), lithium perchlorate (99.99%, Sigma-Aldrich, Germany), acetonitrile (ACN) (99.9%, Merck, Germany), sodium hydroxide (≥98.0%, Merck, Germany), pyrrole (Py) (97.0%, Merck, Germany), orthophosphoric acid (85.0%, Merck, Germany), acetic acid (99.7%, Merck, Germany), boric acid (99.5%, Merck, Germany), ascorbic acid (Merck, Germany), caffeine (99.0%, Merck, Germany), and D­(+)-glucose (99.0%, Merck, Germany) of analytical grade were used as received. Acetonitrile is a volatile, flammable, and toxic organic solvent and should be handled in a well-ventilated fume hood using appropriate personal protective equipment. Ultrapure deionized water (18.2 MΩ·cm, Millipore Milli-Q Elix 10 UV Water Purification System, Germany) was used for the preparation of all solutions. Xeljanz tablets (Pfizer) were purchased from a local pharmacy.

### 2.2 Apparatus and Instrumentation

Electrochemical measurements were carried out using an Autolab PGSTAT302N potentiostat/galvanostat (Metrohm Autolab, Utrecht, Netherlands) controlled by NOVA 1.11 software. A conventional three-electrode cell configuration was employed for all experiments. A 0.7 mm HB pencil lead (Tombow, Japan) was used as the working electrode, while an Ag/AgCl (3 M KCl) electrode and a platinum wire served as the reference and counter electrodes, respectively. The surface morphologies of the bare and modified electrodes were examined using scanning electron microscopy (SEM) (FEI Quanta FEG 250, USA). Fourier Transform Infrared (FT-IR) spectra were recorded using a JASCO FT/IR-4700 spectrometer equipped with an ATR PRO ONE diamond attenuated total reflectance accessory. The analyses were performed in the wavenumber range of 400 to 4000 cm^–1^ with a resolution of 4 cm^–1^ and an accumulation of 32 scans to ensure a high signal-to-noise ratio. Electronic absorption spectra for titration studies were recorded using a Shimadzu UV-1700 PharmaSpec Spectrophotometer (Kyoto, Japan). The measurements were conducted using matched quartz cuvettes with a 1.0 cm path length in the wavelength range of 200 to 400 nm. The instrument was controlled, and the data were processed using the associated software to ensure precise determination of the absorption maxima (λ_max_). pH measurements were performed with a Mettler Toledo (Switzerland) multi-pH/ion meter. All experiments were conducted at room temperature (25 ± 2 °C) under ambient laboratory conditions.

### 2.3 Preparation of Modified Electrodes

Pencil graphite leads (Tombo, HB, 0.7 mm diameter) were cut into 3 cm segments and mounted onto a custom-designed holder. Electrical contact was established by soldering a metal wire to the upper end of each lead. Before modification, the PGEs were thoroughly rinsed with deionized water to remove surface impurities.

The PPy and calix­[n]­arene/PPy modified electrodes were prepared via in situ electropolymerization using the CV technique. The calix­[n]­arene/PPy modified electrodes were fabricated via in situ electropolymerization, as this approach ensures the uniform entrapment of the macrocyclic molecules within the growing conductive polymer matrix. Compared to traditional drop-casting methods, where the modifier may loosely adhere or leach from the surface over time, in situ integration produces a significantly more stable and robust composite layer. Furthermore, the simultaneous growth of the PPy backbone and the calix­[n]­arene units promotes a higher density of active recognition sites that are directly integrated into the polymer matrix, thereby facilitating more efficient host–guest interactions and faster electron transfer kinetics. For the preparation of the PPy/PGE, the precursor solution contained 0.1 M pyrrole and 0.1 M LiClO_4_ in an acetonitrile–water (95:5, v/v) mixture. Electropolymerization was performed by cycling the potential between −0.6 V and +1.2 V at a scan rate of 100 mV s^–1^ for five cycles.

For the calix­[n]­arene/PPy/PGE modifications, 0.005 M of the respective macrocycle (0.0318 g for calix[4]­arene, 0.0478 g for calix[6]­arene, and 0.0637 g for calix[8]­arene) was added to the monomer solution. The electropolymerization process was carried out under the same CV conditions used for the PPy/PGE. The cyclic voltammograms illustrating the systematic growth of the PPy and calix­[n]­arene/PPy films on the PGE surface are presented in Figure 1. Following the modification, all electrodes were gently rinsed with deionized water to remove any unreacted monomers or loosely bound species from the surface.

**Figure 1:**
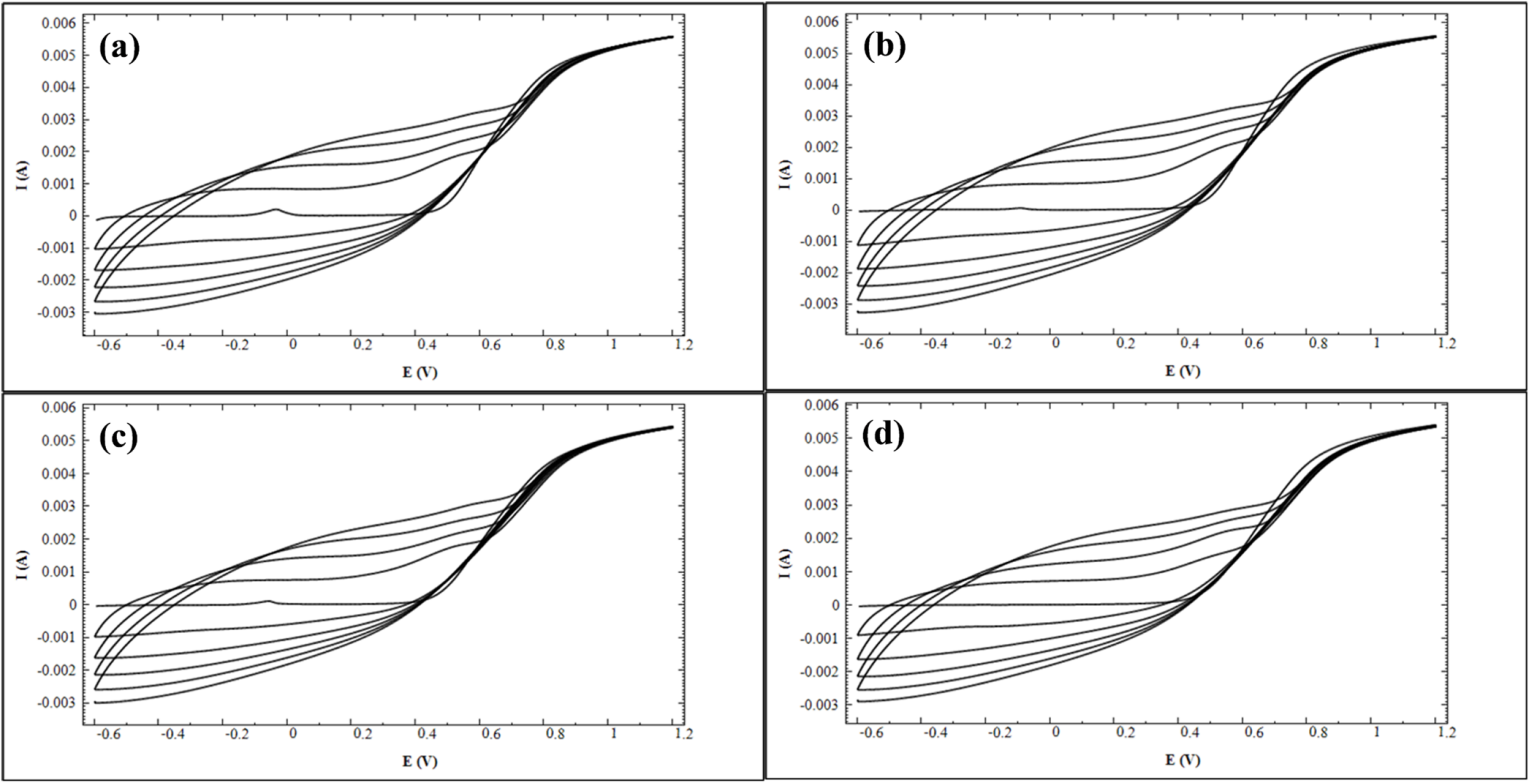
CV voltammogram of the electrodes: (a) PPy/PGE, (b) calix[4]­arene/PPy/PGE, (c) calix[6]­arene/PPy/PGE, and (d) calix[8]­arene/PPy/PGE-modified electrodes.

### 2.4 Voltammetric Measurements

The analytical performance of the sensors was evaluated after optimizing the electrode preparation parameters. Prior to the drug analysis, the electrochemical stability of the PPy and calix­[n]­arene/PPy films was ensured by recording multiple differential pulse voltammograms (DPVs) in a pH 4.0 Britton–Robinson (BR) buffer solution (containing 0.1 M LiClO_4_ in a 10:90 (v/v) acetonitrile–water mixture) until a stable background signal was obtained.

For the quantification of tofacitinib, DPV measurements were performed in the potential range of 0.0 to 1.5 V. All measurements were carried out in triplicate to ensure reproducibility. A 1000 ppm stock solution of tofacitinib was used to prepare working standards ranging from 0.1 to 15 ppm via serial dilution with the BR buffer. Calibration curves were constructed by plotting the resulting anodic peak currents against the respective tofacitinib concentrations.

## 3 Results

### 3.1 Surface Characterization (SEM and EDS)

The surface morphologies of the modified electrodes were examined by SEM to verify the successful formation of the PPy and calix­[n]­arene/PPy films. As shown in Figure 2a, the PPy/PGE surface exhibits the characteristic cauliflower-like globular morphology typical of electropolymerized polypyrrole. This porous and nodular structure provides a high effective surface area, which is crucial for enhanced electrochemical performance.

**Figure 2:**
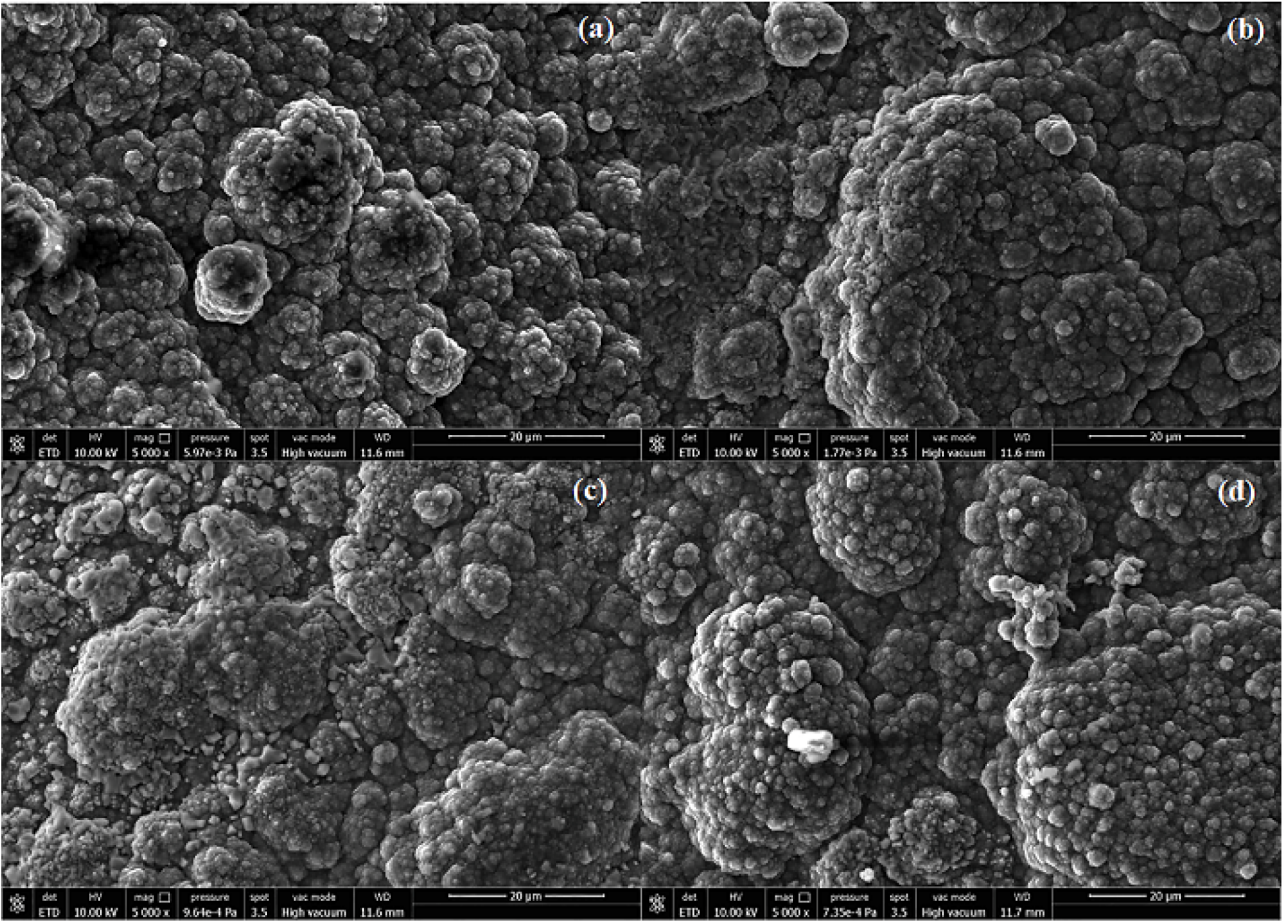
SEM images of the electrode surfaces (20 μm): (a) PPy/PGE, (b) calix[4]­arene/PPy/PGE, (c) calix[6]­arene/PPy/PGE, and (d) calix[8]­arene/PPy/PGE.

Upon the incorporation of calix­[n]­arene derivatives (Figure 2b, c, and d), notable changes in surface topography were observed. The calix­[n]­arene/PPy/PGE surfaces appear more compact and exhibit a more uniform distribution of microglobules compared to the PPy/PGE. Specifically, as the cavity size of the calix­[n]­arene increases from [4] to [8], the surface becomes increasingly dense with finer granular structures. This suggests that the macrocyclic molecules act as dopants during the growth of the PPy chain, influencing the nucleation and growth kinetics of the polymer film.

The integration of these macrocycles not only alters the physical appearance but also introduces supramolecular recognition sites within the polymer matrix. The increased surface roughness and the presence of well-distributed calixarene cavities are expected to facilitate the preconcentration of tofacitinib via host–guest interactions, consistent with the observed enhancement in voltammetric response.

The surface morphologies and elemental compositions of the modified electrodes were investigated to confirm the successful fabrication of the sensor platform. As discussed in the morphological evaluation (Figure 2), the transition from the typical cauliflower-like structure of PPy to a more compact, nodular topography in the presence of calix­[n]­arenes suggested a structural integration. To quantitatively validate this integration, SEM–EDS analysis was performed, and the elemental weight percentages are summarized in Table 1.

**Table 1:** Atomic Compositions of the PPy/PGE, Calix[4]­arene/PPy/PGE, Calix[6]­arene/PPy/PGE, and Calix[8]­arene/PPy/PGE Electrodes^a^

	PPy/PGE		Calix[4]arene/PPy/PGE		Calix[6]arene/PPy/PGE		Calix[8]arene/PPy/PGE	
Element	%Atomic	%Weight	%Atomic	%Weight	%Atomic	%Weight	%Atomic	%Weight
C	54.96	47.07	61.28	52.57	65.84	56.36	58.30	49.23
N	18.00	17.98	19.32	19.33	15.14	15.11	19.44	19.14
O	24.08	27.48	15.14	17.30	14.09	16.07	17.43	19.60
Cl	2.95	7.46	4.26	10.79	4.93	12.46	4.83	12.03

a Not detected.

The EDS spectrum of the PPy/PGE (Figure 3a) confirms the presence of carbon (C) and nitrogen (N), consistent with the polypyrrole backbone (C_8_H_6_N_2_). The detected chlorine (Cl) peak (7.46 wt %) and oxygen (O) peak (27.48 wt %) are attributed to the ClO_4_^–^ dopant ions from the supporting electrolyte used during electropolymerization.

**Figure 3:**
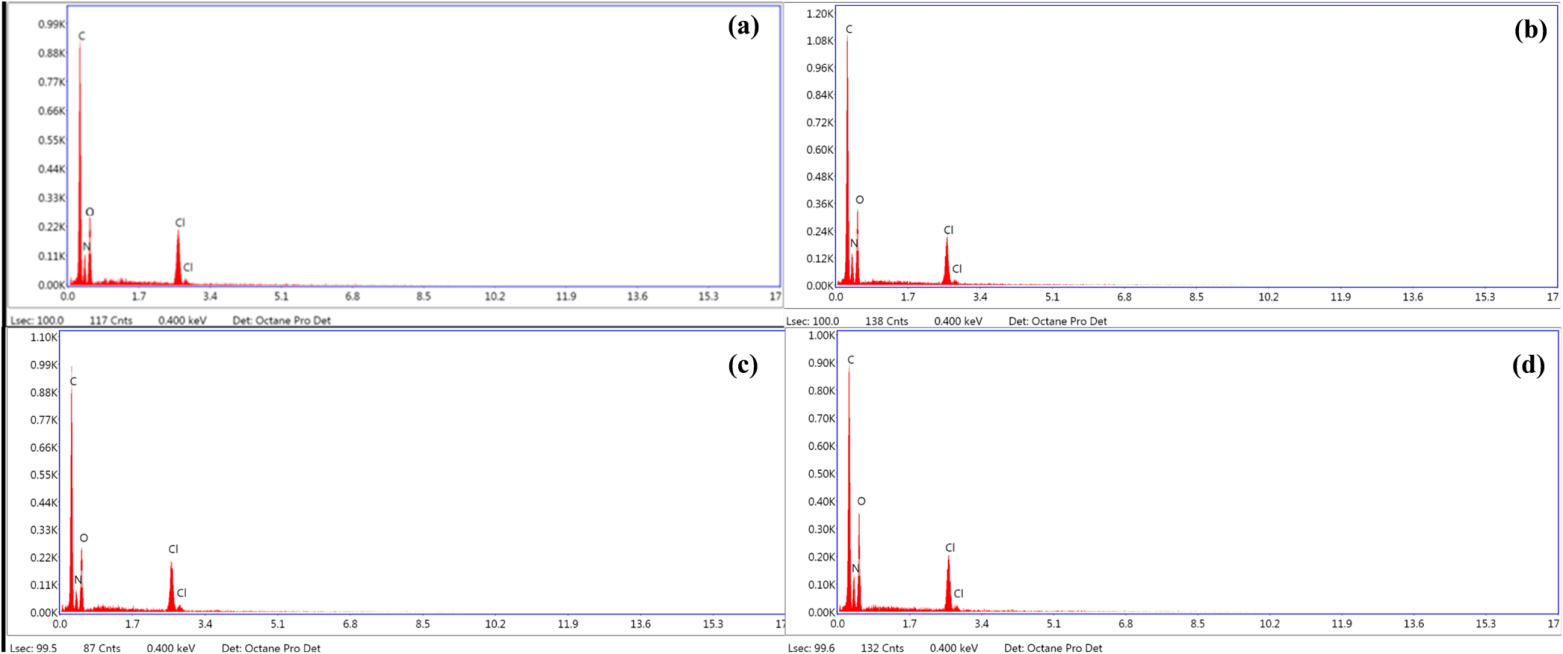
SEM–EDS spectra of the electrodes: (a) PPy/PGE, (b) calix[4]­arene/PPy/PGE, (c) calix[6]­arene/PPy/PGE, and (d) calix[8]­arene/PPy/PGE-modified electrodes.

Crucially, the presence of oxygen was confirmed across all samples. While the oxygen signal in the unmodified PPy/PGE (Figure 3a) is primarily attributed to the high stoichiometric oxygen density of the ClO_4_^–^ dopant ions, the spectra of the calix-modified electrodes (Figure 3b–d) reflect the integrated contribution of both the dopants and the oxygen-containing functional groups of the macrocycles. Following the incorporation of calix­[n]­arenes, a systematic shift in the elemental ratios was observed; while the absolute carbon content increased due to the phenolic architecture of the macrocyclic additives, a concomitant relative decrease in the oxygen weight percentage (from 27.48 wt % to 16.07–19.60 wt %) was recorded.

This phenomenon is ascribed to a “matrix dilution effect” where the significantly increased carbon density within the composite matrix effectively attenuates the relative weight contribution of the oxygen atoms. Among the modified electrodes, the oxygen content reached its highest value at 19.60 wt % for the calix[8]­arene/PPy/PGE, suggesting that this derivative provides the highest density of oxygen-rich interaction sites for tofacitinib recognition. This elemental transition confirms the successful integration of the organic macrocycles into the PPy framework and underscores the altered stoichiometric balance of the modified surface.

These results confirm that the calix­[n]­arene compounds were effectively integrated into the electrode surface during the modification process and further validate the successful fabrication of the composite electrodes.

### 3.2 Optimization Studies

#### 3.2.1 Effect of the Number of Polymerization Cycles

The influence of the number of electropolymerization cycles on the sensor’s performance was investigated to determine the optimal film thickness for tofacitinib determination. PPy/PGE, calix[4]­arene/PPy/PGE, calix[6]­arene/PPy/PGE, and calix[8]­arene/PPy/PGE were prepared using cycle numbers ranging from 2 to 12. As shown in Figure 4, the peak current responses for 8 ppm tofacitinib reached their maximum at 5 cycles for all modified electrodes, which aligns with previous reports in the literature.^17–19^

**Figure 4:**
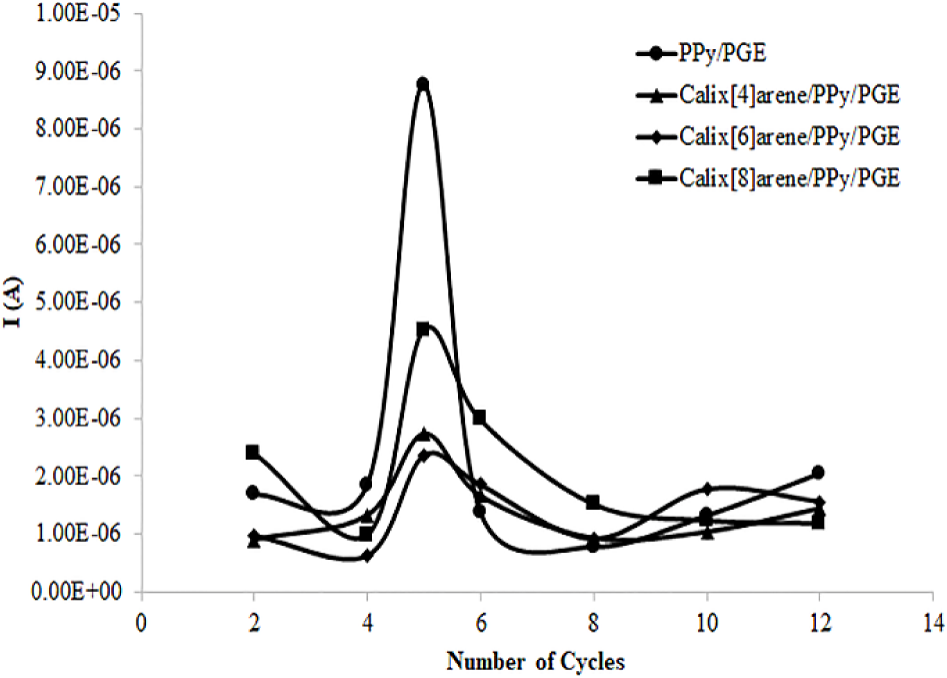
Influence of the number of electropolymerization cycles on the peak current response of 8 ppm tofacitinib.

The number of cycles directly affects the thickness and effective surface area of the conductive polymer matrix. While an initial increase up to 5 cycles provides a stable platform with a high density of calix­[n]­arene recognition sites, further increases lead to an excessively thick and dense PPy film. This thick layer likely reduces the number of accessible active sites and increases the diffusion path for tofacitinib molecules, thereby hindering the electron transfer kinetics at the electrode interface. Consequently, 5 cycles were adopted as the optimum parameter for all subsequent electrode fabrications.

#### 3.2.2 Effect of Scan Rate on the Electrode Response

The influence of the scan rate used during the electropolymerization process on the final electrochemical performance of the sensors was investigated. PPy/PGE and calix­[n]­arene/PPy/PGE electrodes were fabricated at various scan rates to evaluate their response toward 8 ppm tofacitinib. As illustrated in Figure 5, the scan rate directly affects the nucleation and growth kinetics of the PPy film, leading to distinct differences in peak current intensities.

**Figure 5:**
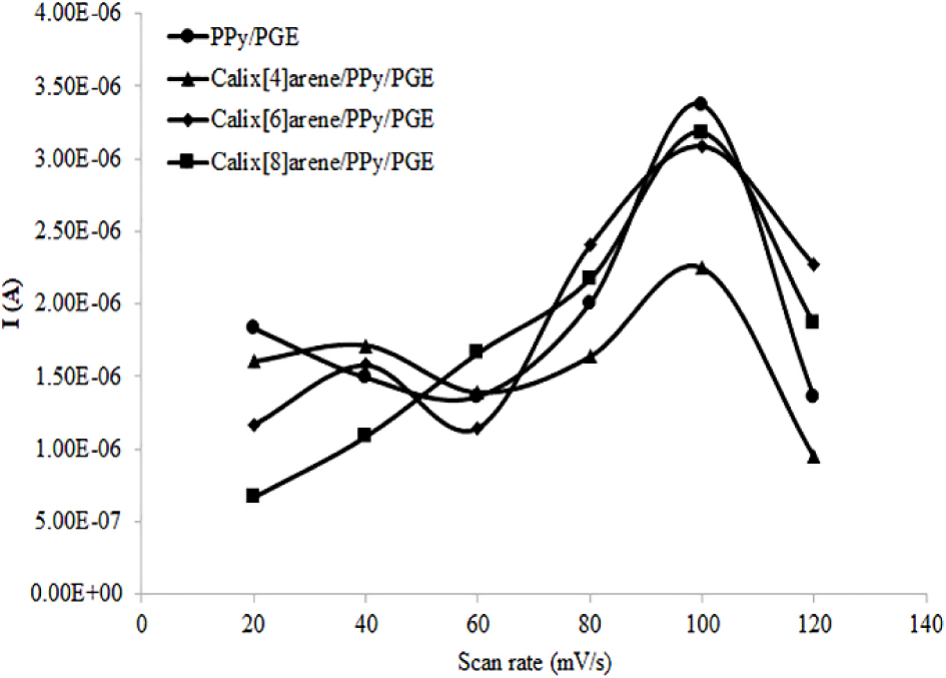
Effect of scan rate during electropolymerization on the electrode response for tofacitinib.

It was observed that a scan rate of 100 mV s^–1^ provided the optimal voltammetric response. At higher scan rates, the formation of a more porous and electrochemically active film likely facilitates the flux of tofacitinib toward the macrocyclic recognition sites. Conversely, very low scan rates might result in overly dense polymer growth that limits mass transport. The selection of 100 mV s^–1^ as the optimal scan rate is in good agreement with previous studies.^18–20^

#### 3.2.3 Effect of Calix­[n]­arene Concentrations on Electrode Response

The PPy/PGE, calix[4]­arene/PPy/PGE, calix[6]­arene/PPy/PGE, and calix[8]­arene/PPy/PGE electrodes were prepared by the CV method at the optimal scan rate of 100 mV/s and the optimal number of cycles (5 cycles) using monomer concentrations of 0.0025, 0.005, 0.01, and 0.015 M. Voltammograms were obtained using DPV analysis with the prepared electrodes, and the maximum peak currents were compared.

The results obtained are given in Figure 6. The peak current increased with calix­[n]­arene concentration up to 0.005 M, indicating an increase in the number of host–guest recognition sites on the electrode surface. However, a decrease in the current response was observed at higher concentrations (0.01 and 0.015 M). This could be attributed to the potential saturation of the PPy matrix with macrocyclic molecules, which may hinder the electropolymerization process or lead to an increase in the nonconductive nature of the film, thereby limiting the electron transfer rate.

**Figure 6:**
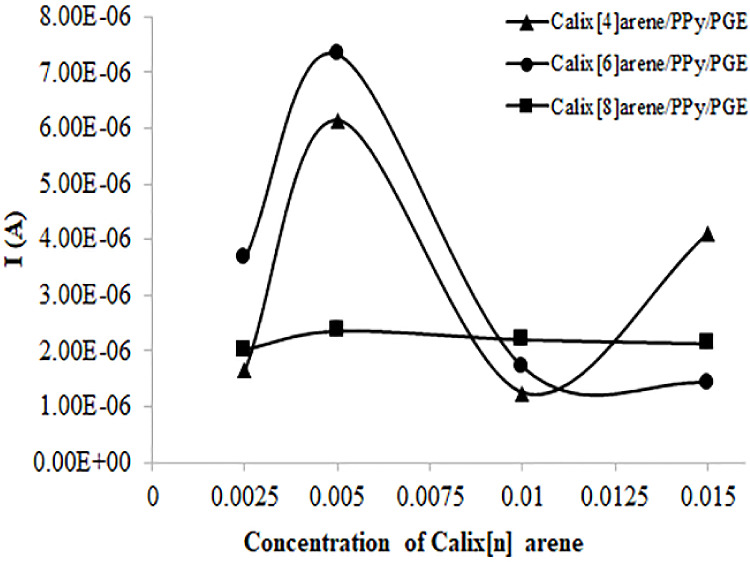
Effect of calix­[n]­arene concentrations on the electrode response.

#### 3.2.4 Effect of Solution pH on the Electrode Response

The pH of the supporting electrolyte plays a vital role in electrochemical sensing, as it affects both the ionization state of the analyte and the electron transfer kinetics. The voltammetric response of 8 ppm tofacitinib was evaluated using PPy/PGE and calix­[n]­arene/PPy/PGE electrodes across a pH range of 2.0 to 8.0 in BR buffer solutions. As shown in Figure 7, the peak current reached its maximum value at pH 4.0 for all modified electrodes, followed by a gradual decrease at higher pH values.

**Figure 7:**
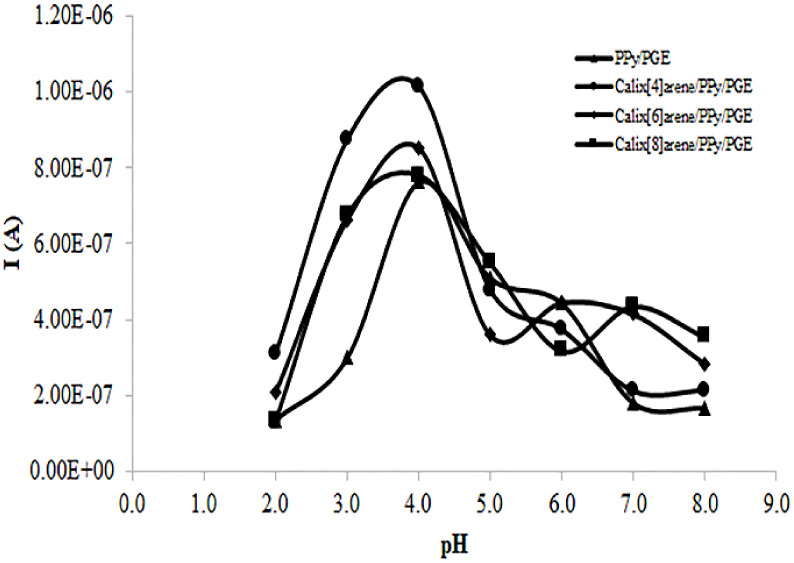
Effect of solution pH on the performance of the electrodes.

The selection of pH 4.0 as the optimum medium is consistent with the ionization behavior of tofacitinib, which possesses p*K*_a_ values of approximately 3.1 and 5.1. In a medium of pH 4.0, tofacitinib exists in a predominantly monoprotonated state. This ionization state facilitates strong supramolecular interactions with the calix­[n]­arene macrocycles via a combination of hydrogen bonding and cation-π interactions within the macrocyclic cavity. While some studies in the literature, such as those utilizing GCE or BDDE, have reported slightly higher pH values (e.g., pH 4.7 or 5.0) as optimal for tofacitinib determination,^15^ our findings indicate that pH 4.0 provides a superior environment for our specific PPy/PGE-based system. This is further supported by the fact that the PPy film maintains higher conductivity in more acidic media, whereas its conductivity diminishes as the environment becomes more alkaline (pH > 6.0).

### 3.3 Spectroscopic Investigation of Host–Guest Interactions

#### 3.3.1 FT-IR Spectroscopic Analysis

FT-IR-ATR spectroscopy was performed to provide direct structural evidence of the host–guest interactions between tofacitinib and calix­[n]­arenes. For this purpose, 100 μM stock solutions of each component were prepared in a 95:5 (v/v) acetonitrile–water mixture, and these solutions were subsequently utilized to record the FT-IR-ATR spectra. The FT-IR-ATR spectra of pure tofacitinib citrate, pure calix­[n]­arene derivatives, and their corresponding 1:1 host–guest mixtures are illustrated in Figures 8–10.

**Figure 8:**
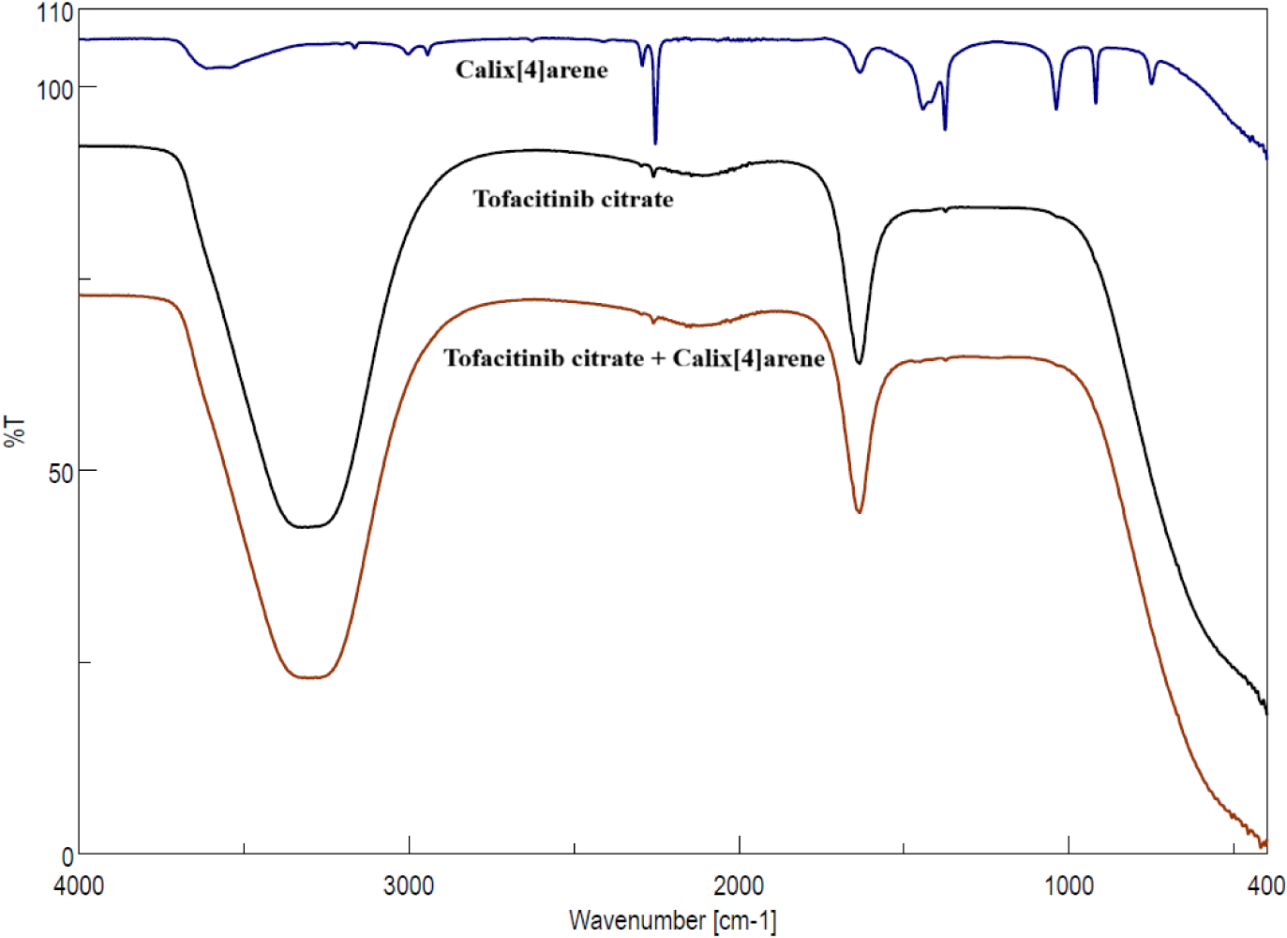
FT-IR-ATR spectra of pure tofacitinib citrate, pure calix[4]­arene, and the tofacitinib citrate with calix[4]­arene mixture.

**9:**
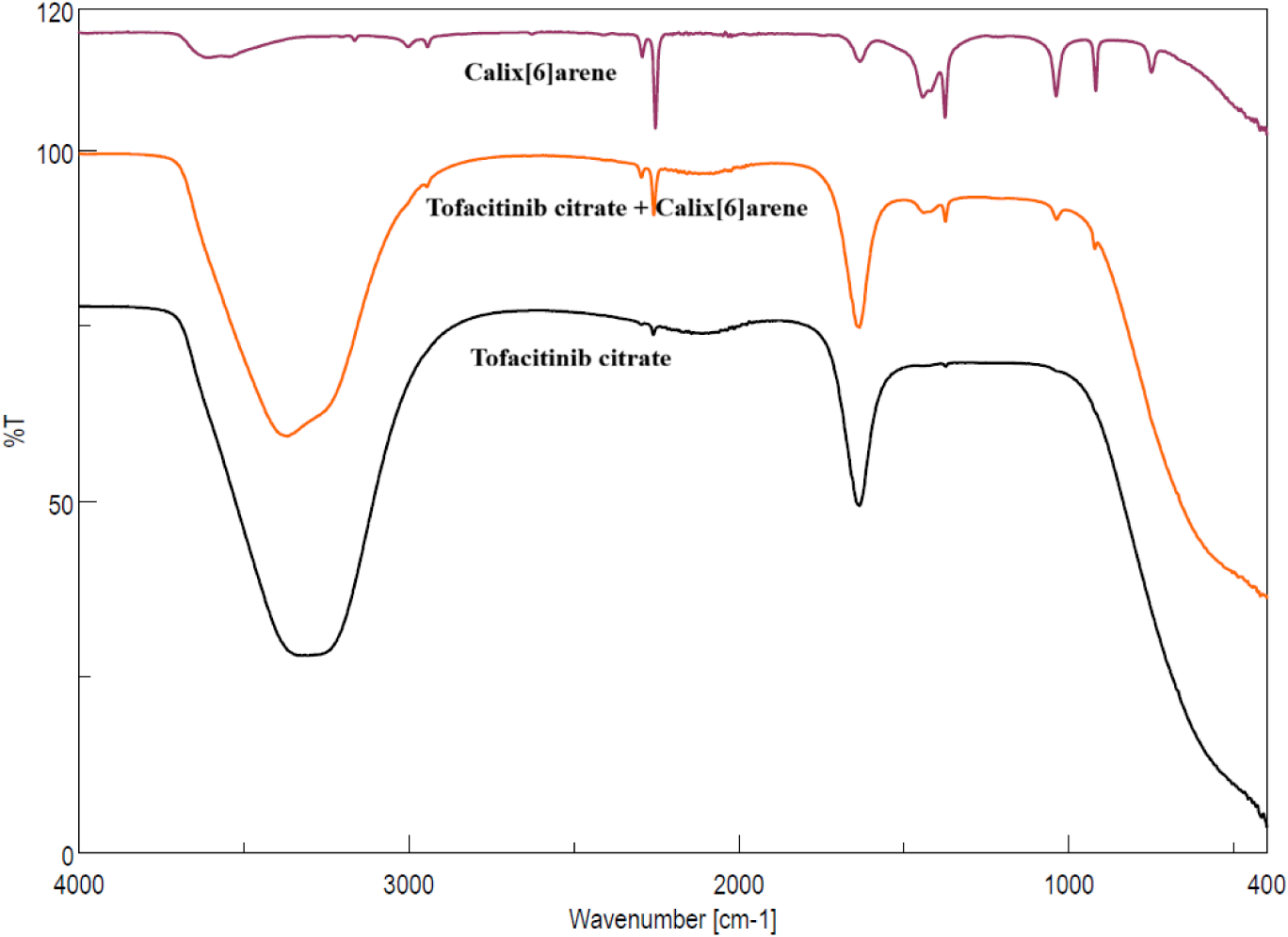
FT-IR-ATR spectra of pure tofacitinib citrate, pure calix[6]­arene, and the tofacitinib citrate with calix[6]­arene mixture.

**Figure 10:**
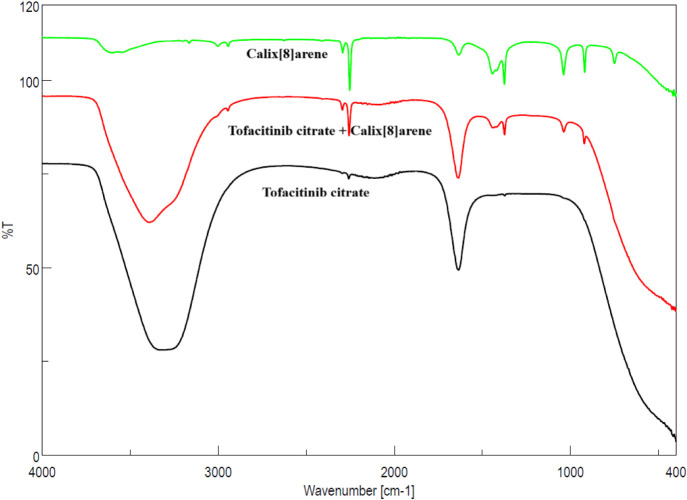
FT-IR-ATR spectra of pure tofacitinib citrate, pure calix[8]­arene, and the tofacitinib citrate with calix[8]­arene mixture.

The FT-IR-ATR spectrum of pure tofacitinib citrate displays a characteristic broad band at 3324.68 cm^–1^, which corresponds to the stretching vibrations of the secondary amine (N–H) and the hydroxyl (O–H) groups of the citrate moiety. The most definitive marker for the drug, the sharp nitrile (C≡N) stretching peak, appears at 2259.2 cm^–1^. In the spectra of pure calix­[n]­arenes (n = 4, 6, 8), broad bands associated with intramolecular hydrogen bonding are observed between 3150 and 3300 cm^–1^, accompanied by the phenolic C–O stretching vibrations near 1038 cm^–1^. The peak observed at 1634.38 cm^–1^ is a crucial spectral marker for tofacitinib citrate, primarily attributed to the stretching vibrations of the tertiary amide carbonyl (C=O) group and the skeletal vibrations of the pyrimidine ring. In the complexation studies, this band remains consistently present across all mixtures, confirming that the drug molecule maintains its structural integrity upon interacting with the calix­[n]­arene cavities.

Calix­[n]­arene derivatives were mixed with tofacitinib citrate at a 1:1 ratio, and FTIR spectra were obtained. The interaction between tofacitinib and the macrocyclic hosts was found to be strongly dependent on the cavity size. In the calix[4]­arene mixture, the characteristic peaks of the host (within the 1000–1500 cm^–1^ region) are largely masked by the dominant signals of tofacitinib. This masking, combined with a relatively small negative shift of 19 units in the N–H/O–H band (to 3305.39 cm^–1^), indicates a steric mismatch where the narrow cavity limits efficient inclusion.

Conversely, the mixtures with calix[6]­arene and calix[8]­arene demonstrate a clear coexistence of both host and guest signals. The visibility of calixarene-specific skeletal vibrations (e.g., 1037 cm^–1^ and 1440 cm^–1^) in these mixtures confirms a high degree of structural complementarity. For calix[6]­arene, a significant shift of 44 cm^–1^ (3368.07 cm^–1^) was observed, suggesting more favorable encapsulation than the calix[4] derivative.

The most pronounced spectral change occurred with calix[8]­arene, which exhibited a dramatic shift of 66 cm^–1^ (3390.24 cm^–1^). This substantial shift, alongside the clear visibility of host peaks, provides robust physical evidence that the largest cavity offers the most ideal environment for the full incorporation of Tofacitinib. The systematic increase in these shifts (calix[4] < calix[6] < calix[8]) perfectly correlates with the electrochemical sensitivity results. It confirms that the larger cavities facilitate easier molecular entry and maximize the interaction surface area, leading to the superior preconcentration and sensitivity observed in the voltammetric measurements (8 > 6 > 4).

#### 3.3.2 UV–Vis Titration and Binding Affinity

To complement the FT-IR findings, further elucidate the nature of the supramolecular interactions, and quantitatively assess the binding affinity between tofacitinib and the different calix­[n]­arene derivatives, UV–vis titration studies were conducted. The experimental data were analyzed using the Benesi–Hildebrand model to determine the binding constants (*K*_*a*_).

The spectroscopic titration experiments were performed using stock solutions (100 μM) of each component prepared in an acetonitrile–water (95:5, v/v) mixture. Initially, the absorbance of a 10 μM calix­[n]­arene solution was recorded as the baseline. Subsequently, increasing concentrations of tofacitinib (5, 10, 20, 30, and 40 μM) were added to the calix­[n]­arene solution, and the resulting absorbance spectra were measured. To determine the binding constants (*K*_*a*_), the data were analyzed using the Benesi–Hildebrand method by plotting the reciprocal of the change in absorbance (1/ΔA) against the reciprocal of the tofacitinib concentration (1/[Tofacitinib citrate]). The *K*_*a*_ values (in M^–1^) were then calculated from the ratio of the intercept to the slope (n/m) obtained from the resulting linear regression equations (y = mx+n), incorporating a 10^6^ conversion factor to account for the initial micromolar (μM) concentrations.

The spectroscopic data presented in Table 2 confirm the existence of supramolecular interactions between tofacitinib and the calix­[n]­arene derivatives. The negative intercept observed in the Benesi–Hildebrand plot for calix[4]­arene suggests that the 1:1 host–guest inclusion model is physically inconsistent for this specific derivative. This deviation is attributed to the relatively small and rigid cavity size of calix[4]­arene (approximately 3.0 Å), which imposes significant steric hindrance for the encapsulation of the bulky tofacitinib molecule. Consequently, the interaction between calix[4]­arene and tofacitinib is likely limited to nonspecific surface adsorption rather than a stable, well-defined inclusion complex, leading to its lower analytical performance compared to the larger calix[8]­arene.

**Table 2:** Benesi–Hildebrand (B–H) Plot Parameters and Binding Constants (*K_a_*) for the Interaction of Tofacitinib with Different Calix­[n]­arenes

Host	λmax (nm)	Δλ (nm)	B–H Equation (y = mx+n)	r^2^	*K*_*a*_ (M^–1^)
Calix[4]	274 →283	9	y = 70.809x – 0.21	0.9996	n.a.^a^
Calix[6]	283 →282	1	y = 55.654x + 0.047	0.9996	844.5
Calix[8]	271 →287	16	y = 56.854x + 0.111	0.9998	1952.4

a n.a.: Not applicable due to a negative intercept, indicating weak or nonspecific binding not conforming to the 1:1 model.

Calix­[8]­arene exhibited the highest binding constant (*K*_*a*_ = 1952.4 M^–1^) and the most significant bathochromic shift (16 nm), clearly identifying it as the most effective host for tofacitinib. The higher binding constant of calix[8]­arene compared to calix[6]­arene (*K*_*a*_ = 844.5 M^–1^) proves that the spatial and electronic environment of tofacitinib is most optimally accommodated within the larger calix[8] cavity, forming a highly stable 1:1 complex.

Crucially, the recognition mechanism in the proposed sensor platform is a “surface-confined” process involving calixarenes entrapped within the conductive PPy matrix. While the binding constants obtained in a free organic solvent system (MeCN/H_2_O) provide essential thermodynamic insights, they may not fully represent the host–guest dynamics within the restricted polymeric microenvironment of the electrode surface. Nevertheless, the superior spectroscopic response and the highest *K*_*a*_ value of calix[8]­arene are in excellent agreement with the electrochemical results, which yielded the highest sensitivity and the lowest LOD values (0.0184 ppm). This convergence of spectroscopic and electrochemical data confirms that the calix[8]­arene-modified PPy/PGE system provides the most ideal platform for the sensitive determination of tofacitinib.

### 3.4 Analytical Performance for Tofacitinib Determination

Under the established optimal conditions, the performance of the developed sensors was evaluated using the DPV technique for tofacitinib citrate concentrations ranging from 0.1 to 15 ppm. A significant enhancement in the peak current response was observed for the calix­[n]­arene/PPy/PGE electrodes compared to the unmodified PPy/PGE, highlighting the superior electrocatalytic and preconcentration capabilities of the macrocyclic derivatives.

The analytical parameters, including the linear range, correlation coefficient (r^2^), limit of detection (LOD), and limit of quantification (LOQ), are summarized in the following sections. The LOD and LOQ were calculated based on the following equations:

$$\mathrm{LOD}=\frac{3.3s}{m}$$

and

$$\mathrm{LOQ}=\frac{10s}{m}$$

where *s* represents the standard deviation of the intercept, and *m* is the slope of the analytical calibration curve (Figure 11).

**Figure 11:**
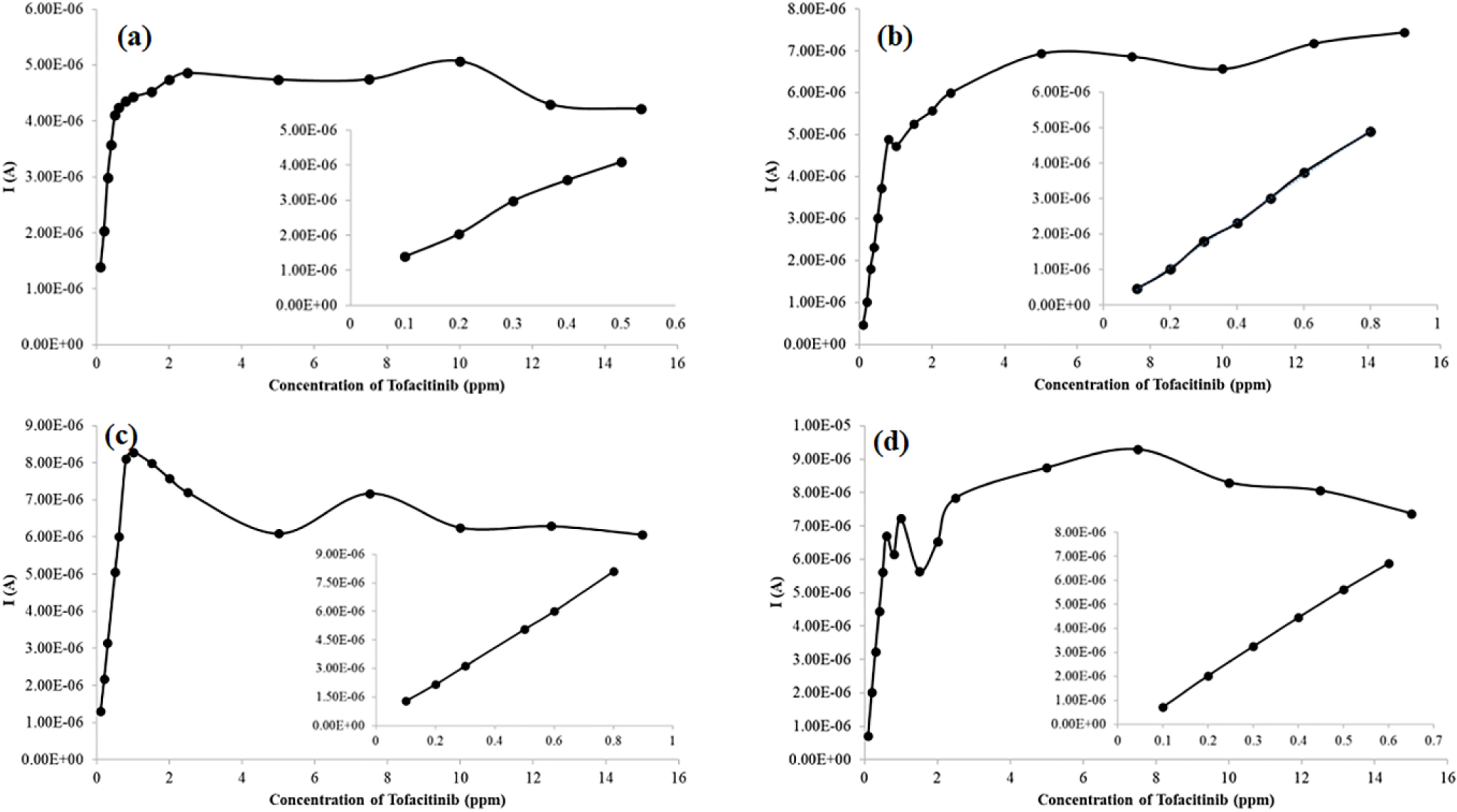
Calibration plots obtained by the DPV method: (a) PPy/PGE, (b) calix[4]­arene/PPy/PGE, (c) calix[6]­arene/PPy/PGE, and (d) calix[8]­arene/PPy/PGE electrodes.

The analytical performance of the developed sensors highlights the significant impact of each modification on tofacitinib determination. For the unmodified PPy/PGE, a linear range of 0.1–0.5 ppm was observed (r^2^ = 0.9991) with a LOD of 0.0271 ppm. The integration of macrocyclic derivatives led to a distinct improvement in sensor responses; the calix[4]­arene/PPy/PGE exhibited a linear working range of 0.1–0.8 ppm (r^2^ = 0.9992) and an LOD of 0.0275 ppm. Continuing the series, the calix[6]­arene/PPy/PGE reached an LOD of 0.0212 ppm with high correlation (r^2^ = 0.9996) over a 0.1–0.8 ppm range. The highest sensitivity was achieved with the calix[8]­arene/PPy/PGE, yielding the lowest LOD of 0.0184 ppm within a linear range of 0.1–0.6 ppm (r^2^ = 0.9996).

The observed decrease in LOD values from calix[4] to calix[8] indicates that the analytical performance is directly governed by the macrocyclic ring size, where Calix[8]­arene offers the most favorable spatial orientation and electronic environment for tofacitinib recognition. The systematic enhancement in sensitivity from calix[4] to calix[8] is not merely a function of surface area but a result of the superior host–guest complementarity. Tofacitinib, with its bulky pyrrolo­[2,3-*d*]­pyrimidine framework, requires a more expansive macrocyclic architecture for effective inclusion. Calix[8]­arene, having a larger cavity diameter (approximately 9.5–11.7 Å) compared to calix[4]­arene (approximately 3.0 Å), provides the necessary spatial orientation to encapsulate the analyte while maximizing π-π interactions between the drug’s aromatic rings and the phenolic scaffold of the macrocycle. The linear working range of 0.1–0.8 ppm demonstrates the high sensitivity of the proposed electrode and confirms its suitability for the determination of pharmaceutical active ingredients in both commercial formulations and urine samples at low ppm levels commonly encountered in these matrices.

The DP voltammograms recorded for PPy/PGE and the three calix­[n]­arene-modified electrodes across their respective linear concentration ranges are presented in Figure 12. As illustrated, a systematic enhancement in the anodic peak current was observed with increasing tofacitinib concentrations for all electrode types. Specifically, the voltammograms obtained for PPy/PGE (Figure 12a), calix[4]­arene/PPy/PGE (Figure 12b), calix[6]­arene/PPy/PGE (Figure 12c), and calix[8]­arene/PPy/PGE (Figure 12d) clearly show that the oxidation peak potential remains stable at approximately +0.7 V. This stability confirms the high reproducibility of the modified electrode surfaces and a consistent electrochemical oxidation mechanism throughout the analysis.

**Figure 12:**
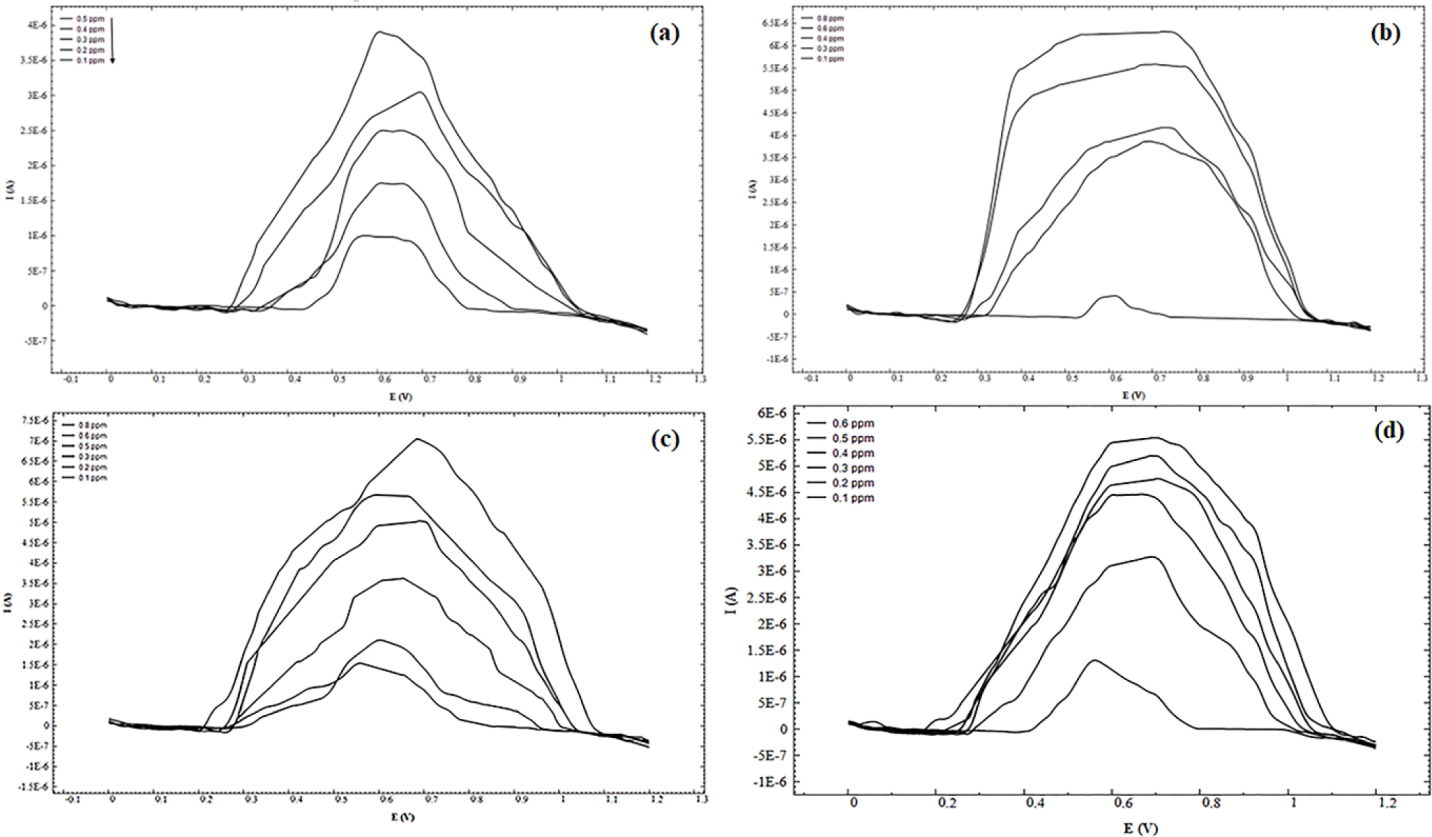
DP voltammograms of tofacitinib obtained by the DPV method at the (a) PPy/PGE, (b) calix[4]­arene/PPy/PGE, (c) calix[6]­arene/PPy/PGE, and (d) calix[8]­arene/PPy/PGE electrodes.

The calibration plots derived from these voltammograms demonstrate excellent linearity, which is essential for the reliable quantification of the drug in real matrices. As shown in Figure 12d, the calix[8]­arene/PPy/PGE achieved the highest peak currents and the most sensitive response compared to the other modifications. The significantly sharper and more sensitive response observed for the calix[8]­arene/PPy/PGE compared to other modifications is attributed to its optimized macrocyclic cavity size. This structure facilitates the effective preconcentration of tofacitinib at the electrode interface through superior host–guest interactions.

The influence of electrode modification on the electrochemical detection of 0.3 ppm tofacitinib citrate was investigated using DPV in BR buffer solution (pH 4). DPV results showed peak currents of 2.5183 × 10^–6^ A for the unmodified PPy/PGE, which increased to 3.9774 × 10^–6^, 4.4171 × 10^–6^, and 4.5044 × 10^–6^ A for the calix[4], [6], and [8] derivatives, respectively. As illustrated in Figure 13, all calix­[n]­arene-modified electrodes exhibited superior current responses compared to the bare PPy/PGE, demonstrating that the integration of these macrocycles effectively amplifies the electrochemical signal. This overall improvement is attributed to the presence of calixarene cavities at the electrode interface, which provide specialized sites for host–guest recognition and facilitate the preconcentration of tofacitinib. While the enhancement was observed across all derivatives, the progressively higher currents recorded for the [6] and [8] versions suggest that larger cavity sizes further optimize the spatial fit and interaction efficiency for the analyte.

**Figure 13:**
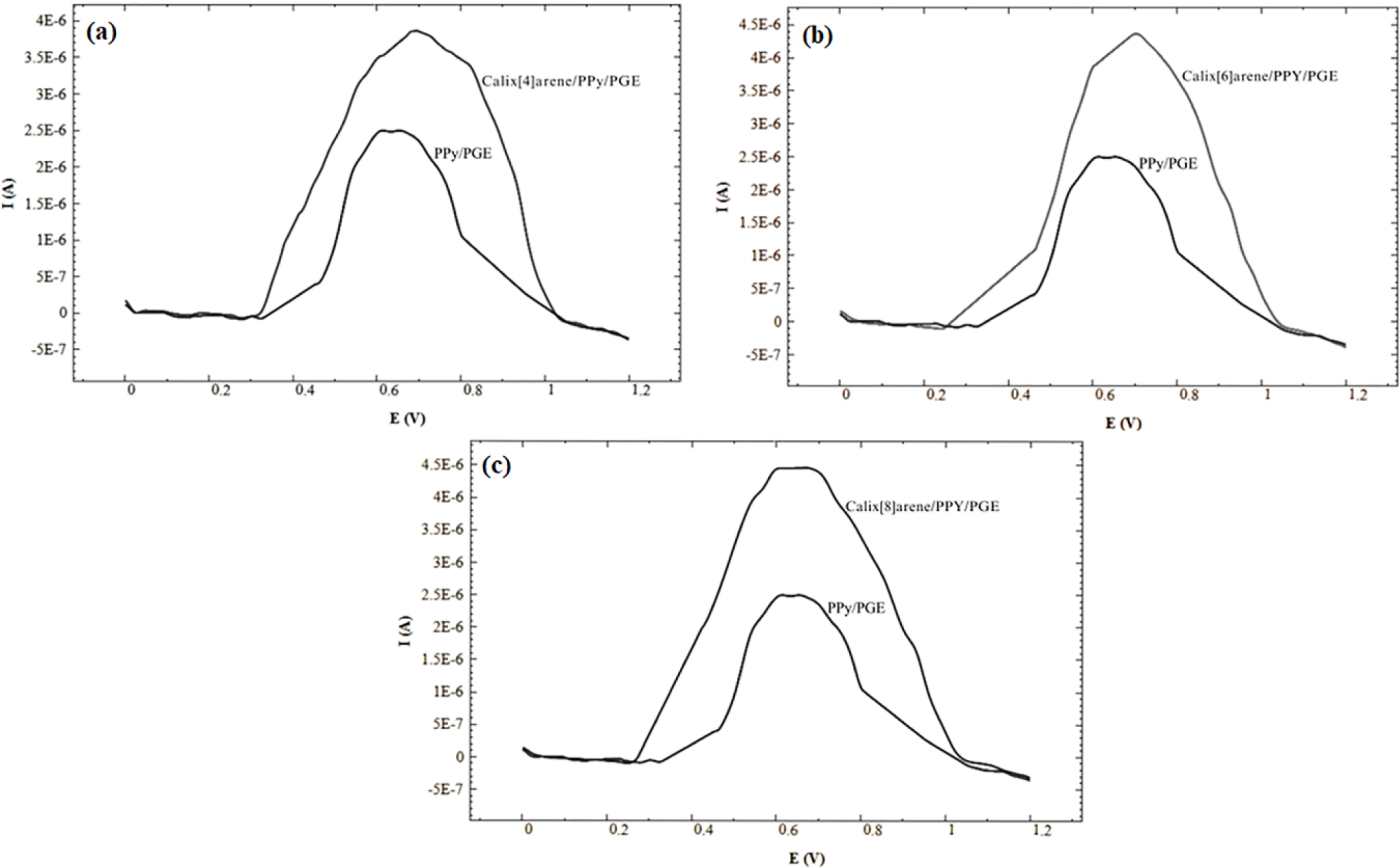
Comparison of DP voltammograms for the determination of 0.3 ppm tofacitinib citrate using different electrodes: (a) calix[4]­arene/PPy/PGE, (b) calix[6]­arene/PPy/PGE, and (c) calix[8]­arene/PPy/PGE.

### 3.5 Selectivity and Interference Studies

The selectivity of the proposed sensors is a vital parameter for their practical application in complex biological and pharmaceutical matrices. The electrochemical response of the calix­[n]­arene/PPy/PGE electrodes toward 0.6 ppm tofacitinib citrate was investigated in the presence of common interfering species, including ascorbic acid, caffeine, and glucose, at concentration levels ranging from 0.2 to 1.2 ppm. The resulting changes in the DPV peak currents are summarized in Table 3.

**Table 3:** Effect of Interfering Species on the DPV Current Response of Calix­[n]­arene-Modified PGEs to 0.6 Ppm Tofacitinib Citrate

		Change in Current Response (μA) (Percent Change %)		
Interfering Species	Concentration (ppm)	Calix[4]arene/PPy/PGE	Calix[6]arene/PPy/PGE	Calix[8]arene/PPy/PGE
Ascorbic acid	0.2	+1.5822 (42.42)	+1.4182 (23.61)	+0.4349 (6.49)
	0.4	+1.7743 (47.57)	+2.1456 (35.72)	+0.7315 (10.92)
	0.6	+1.8760 (50.30)	+3.1656 (52.70)	+1.5458 (23.08)
	1.2	+2.5509 (68.39)	+3.7548 (62.51)	+2.0089 (29.99)
Caffeine	0.2	+0.1162 (3.12)	+0.0656 (1.09)	+0.8299 (12.39)
	0.4	+0.6696 (17.95)	+0.7177 (11.95)	+1.4242 (21.26)
	0.6	+0.9022 (24.19)	+1.0029 (16.70)	+1.5699 (23.43)
	1.2	+1.1060 (29.65)	+1.4102 (23.48)	+1.8435 (27.52)
Glucose	0.2	+0.0665 (1.78)	+0.0424 (0.71)	+0.6693 (9.99)
	0.4	+0.4932 (13.22)	+0.7507 (12.50)	+1.4236 (21.25)
	0.6	+1.4751 (39.55)	+1.6556 (27.56)	+1.7825 (26.61)
	1.2	+1.9766 (52.99)	+2.1472 (35.75)	+2.1920 (32.72)

According to the results, all three modified electrodes demonstrated remarkable stability and selectivity against the tested interferents. In particular, the calix[8]­arene/PPy/PGE electrode exhibited the lowest current variation in the presence of ascorbic acid at low concentrations (0.2–0.4 ppm), suggesting a highly selective interaction between the tofacitinib molecules and the n = 8 macrocyclic cavities.

While an increase in the concentration of interfering species to 1.2 ppm (a 2-fold excess relative to the analyte) led to a more noticeable change in the current response, the overall deviations remained within an acceptable range for analytical determination in real samples. The observed high selectivity can be attributed to the specific host–guest recognition capabilities of the calix­[n]­arene framework and the molecular sieving effect of the polypyrrole matrix, which effectively minimizes the influence of coexisting compounds. These findings confirm that the developed calix­[n]­arene-based sensors are suitable for the reliable determination of tofacitinib in the presence of typical physiological and pharmaceutical interferents.

The selectivity of the calix­[n]­arene/PPy/PGE sensors was evaluated by calculating both the absolute current change (μA) and the percentage relative change (%) in the presence of various interferents. As summarized in Table 3, all tested species (ascorbic acid, caffeine, and glucose) resulted in a positive deviation (increase) in the peak current. While higher concentrations of interferents (e.g., 1.2 ppm) led to more significant signal enhancements, particularly with ascorbic acid, the calix[8]­arene/PPy/PGE electrode demonstrated superior resistance to interference compared to the other modifications. The observed increase in current can be attributed to the overlapping oxidation potentials or background contributions of these species. However, it is important to note that for pharmaceutical dosage form analysis, these physiological interferents are typically absent. For clinical applications in complex matrices like urine, the high sensitivity of the developed method allows for significant sample dilution, which effectively reduces the concentrations of interfering species below their threshold of significant impact. These results, combined with the host–guest recognition of the calix­[n]­arene cavities, confirm the practical applicability of the sensors. For clinical samples, a simple sample pretreatment or dilution step can be employed to minimize these effects.

### 3.6 Electrochemical Determination of Tofacitinib Citrate in Urine and Pharmaceutical Samples

The feasibility of the developed calix­[n]­arene/PPy/PGE sensors for clinical and pharmaceutical monitoring was evaluated by determining tofacitinib citrate in human urine and commercial pharmaceutical formulations. The standard addition method was employed to minimize matrix effects and ensure accurate quantification. Recovery values were calculated by spiking the samples with known concentrations of tofacitinib citrate within the established linear calibration range.

#### 3.6.1 Urine Sample Analysis

For the preparation of the biological matrix, 1 mL of urine collected from a healthy volunteer was diluted with 19 mL of deionized water. Subsequently, a 2 mL aliquot of this dilution was treated with 3 mL of ACN to precipitate proteins and other potentially interfering biological components. Finally, 10 mL of pH 4.0 BR buffer was added to the mixture to reach a final volume of 15 mL, ensuring that the optimal pH for detection was maintained.

DPV measurements were performed in triplicate (N = 3) using the calix[4]­arene/PPy/PGE, calix[6]­arene/PPy/PGE, and calix[8]­arene/PPy/PGE electrodes for urine samples spiked with 0.2 and 0.4 ppm tofacitinib citrate. The obtained DPV voltammograms for the urine samples are given in Figure 14. The determination and recovery results are summarized in Table 4.

**Figure 14:**
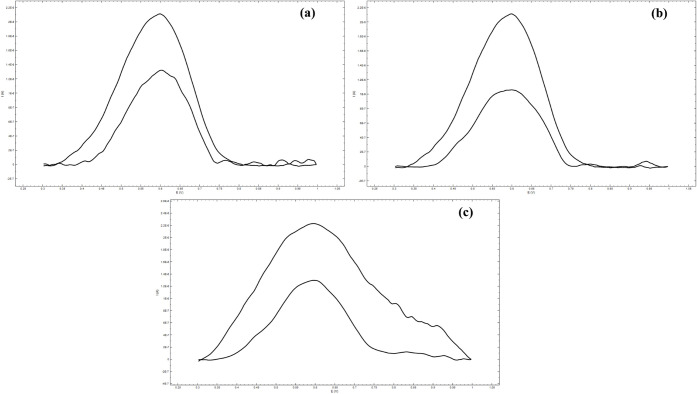
Comparison of DP voltammograms for the determination of 0.2 and 0.4 ppm tofacitinib citrate in urine samples using different electrodes: (a) calix[4]­arene/PPy/PGE, (b) calix[6]­arene/PPy/PGE, and (c) calix[8]­arene/PPy/PGE.

**Table 4:** Determination and Recovery Results of Tofacitinib Citrate in Urine Samples

	Urine Samples							
Electrodes	Spiked Tofacitinib Citrate (ppm)		Determined Tofacitinib Citrate (ppm)		Relative Standard Deviation % (*n* = 3)		Recovery Factor (%)	
Calix[4]arene/PPy/PGE	0.200	0.400	0.203	0.404	1.080	0.672	101.5	101.0
Calix[6]arene/PPy/PGE	0.200	0.400	0.197	0.402	0.317	0.695	98.5	100.5
Calix[8]arene/PPy/PGE	0.200	0.400	0.196	0.402	0.782	1.129	98.0	100.5

The recovery factors were found to be between 98.0% and 101.5%, while the relative standard deviations (RSD) remained below 1.13% for all electrode types. These results indicate that the presence of common urine constituents does not significantly interfere with the sensor response. The successful recovery of tofacitinib in these complex samples further validates that the macrocyclic framework provides excellent selectivity and stability, making it a promising candidate for point-of-care diagnostic applications.

#### 3.6.2 Pharmaceutical Sample Analysis

To evaluate the practical applicability of the calix­[n]­arene/PPy/PGE electrodes for pharmaceutical quality control, a commercial tablet formulation, Xeljanz (Pfizer), containing 5 mg of tofacitinib citrate per tablet, was analyzed. Ten tablets were weighed individually on an analytical balance, and the average weight of a single tablet was calculated by dividing the total weight by the number of tablets. The tablets were then crushed into a fine powder using a clean, smooth mortar and pestle. The resulting powder was transferred onto a clean, white sheet of paper and divided into four equal portions. Equal amounts from each portion were weighed precisely to 0.20718 g on an analytical balance. A total of 100 mL of a 10% ACN-water mixture was added to the weighed powder, and the solution was stirred using a magnetic stirrer. The 50 ppm stock solution prepared from the tablets was used to achieve the final working concentrations (0.2–0.4 ppm) by adding calculated microvolumes (e.g., 60–120 μL) of the stock into the 15 mL electrochemical cell containing the supporting electrolyte.

DPV measurements were performed in triplicate for pharmaceutical samples spiked with 0.2 and 0.4 ppm tofacitinib citrate. The obtained DPV voltammograms for pharmaceutical samples are given in Figure 15. The determination and recovery results are detailed in Table 5. The recovery factors ranged from 98.5% to 101.5%, with relative standard deviations (RSD) remaining below 1.30%. The experimental results obtained from the analysis of Xeljanz tablets were found to be in excellent agreement with the manufacturer’s label claim of 5 mg tofacitinib citrate per tablet, as evidenced by the high recovery percentages shown in Table 5. These results demonstrate that the developed sensors can accurately determine tofacitinib citrate in its commercial form without being affected by common tablet binders or fillers.

**Figure 15:**
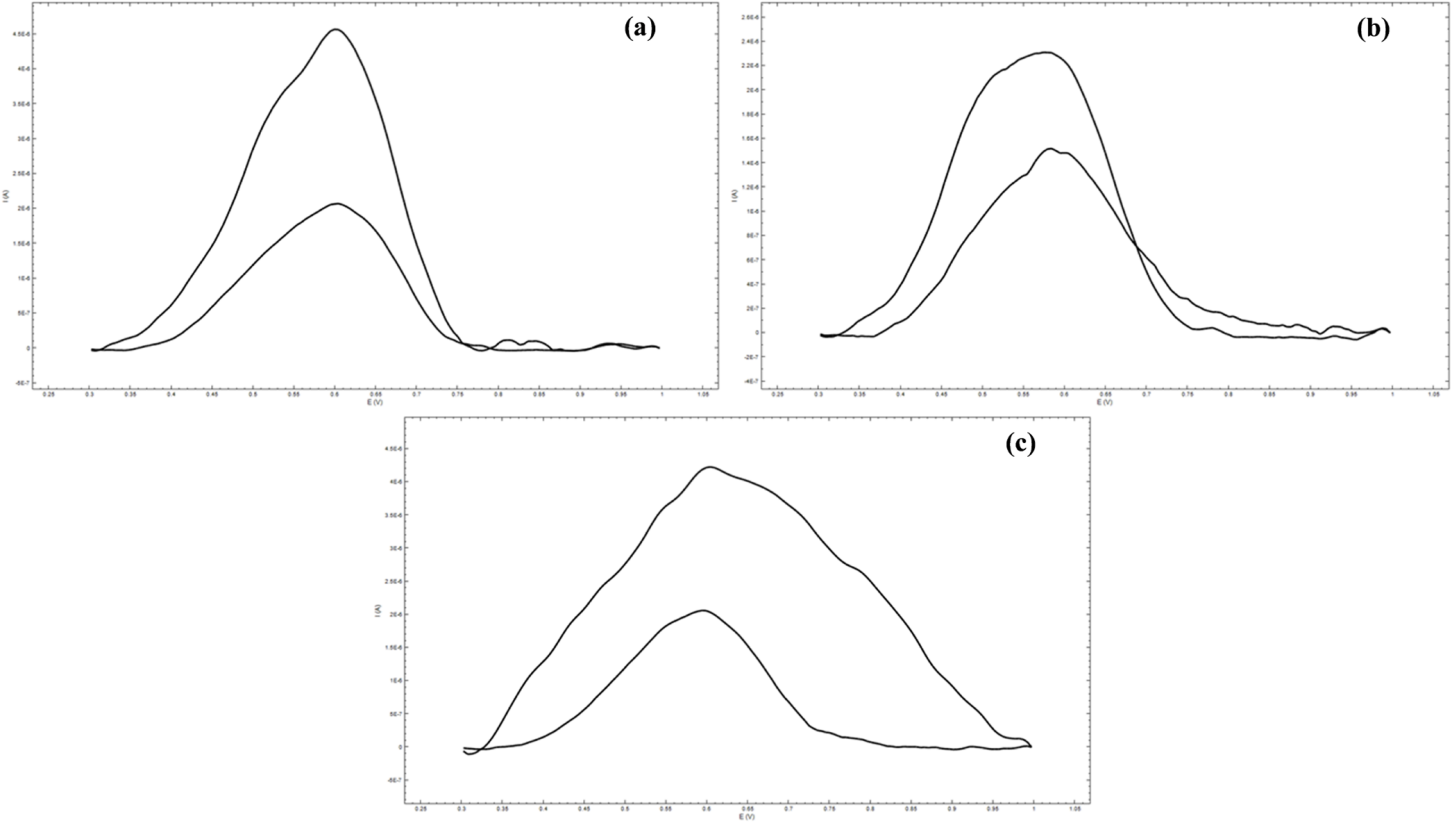
Comparison of DP voltammograms for the determination of 0.2 and 0.4 ppm tofacitinib citrate in pharmaceutical samples using different electrodes: (a) calix[4]­arene/PPy/PGE, (b) calix[6]­arene/PPy/PGE, and (c) calix[8]­arene/PPy/PGE.

**Table 5:** Determination and Recovery Results of Tofacitinib Citrate in Pharmaceutical Samples

Reported Content (Xeljanz)	5^a^							
Electrodes	Spiked Tofacitinib Citrate (ppm)		Determined Tofacitinib Citrate (ppm)		Relative Standard Deviation % (*n* = 3)		Recovery Factor (%)	
Calix[4]arene/PPy/PGE	0.200	0.400	0.199	0.405	1.298	0.663	99.5	101.3
Calix[6]arene/PPy/PGE	0.200	0.400	0.198	0.401	0.756	0.597	99.0	100.3
Calix[8]arene/PPy/PGE	0.200	0.400	0.197	0.406	0.698	0.872	98.5	101.5

a mg per tablet.

## 4 Discussion and Conclusion

In this research, a series of calix­[n]­arene-modified pencil graphite electrodes (calix[4]­arene/PPy/PGE, calix[6]­arene/PPy/PGE, and calix[8]­arene/PPy/PGE) were successfully fabricated through a one-step electropolymerization process. To the best of our knowledge, this study represents the first voltammetric investigation of tofacitinib citrate determination using a calix­[n]­arene-modified PGE platform.

The electrodes were prepared under optimized cyclic voltammetry conditions, specifically within a potential range of −0.6 to +1.2 V, a scan rate of 100 mV/s, and a total of 5 cycles. SEM and EDS analyses confirmed the successful surface modification, revealing distinct nodular morphologies and the characteristic appearance of oxygen signals associated with the phenolic macrocycles.

The analytical performance of the sensors demonstrated a systematic and highly sensitive relationship between the anodic peak current and tofacitinib concentration across all modified surfaces. A key finding of this study is that all calix­[n]­arene-modified electrodes exhibited significantly superior sensitivity, wider linear ranges, and lower detection limits compared to the unmodified PPy/PGE. This collective enhancement confirms that the incorporation of macrocyclic calix­[n]­arene derivatives into the polypyrrole matrix creates a more effective sensing platform, regardless of the specific ring size.

While all derivatives showed excellent performance, the results followed a progressive improvement as the macrocyclic ring size increased from n = 4 to n = 8. The calix[8]­arene/PPy/PGE yielded the most favorable results, achieving a detection limit (LOD) of 0.0184 ppm. This trend suggests that while all calixarene cavities facilitate the preconcentration of tofacitinib via host–guest interactions, the larger n = 8 derivative provides the most optimized spatial and electronic environment for the bulky analyte. The successful application of all three modified electrodes in urine and pharmaceutical samples, with recovery values ranging from 98.0% to 101.5%, further validates the robustness of the calix­[n]­arene-based modification strategy.

In conclusion, the calix­[n]­arene-modified PGE series offers a sensitive, low-cost, and rapid sensing platform for tofacitinib determination. The successful application in both biological and pharmaceutical matrices demonstrates its potential for routine quality control and point-of-care diagnostic monitoring.

As illustrated in Table 6, a comprehensive survey of the literature reveals that the quantification of tofacitinib citrate has been predominantly carried out using HPLC coupled with various detectors.^6,11–14^ While HPLC methods provide high sensitivity and reliability, they often involve expensive equipment, complex sample preparation steps, and the consumption of large volumes of organic solvents. Notably, there is a noticeable scarcity of electrochemical studies for tofacitinib determination, with only a few reports available.^15,16^ This highlights a significant gap in the development of alternative, low-cost, and portable sensing platforms. Our study addresses this gap by offering a highly sensitive, disposable, and rapid electrochemical method, which rivals the sensitivity of standard HPLC techniques while providing the distinct advantages of point-of-care potential and minimal cost.

**Table 6:** Comparison of Analytical Performance of Different Methods for Tofacitinib Determination

Method	Linear Range (ppm)	LOD (ppm)	pH	Sample	Recovery (%)	ref.
UHPLC	2.0 × 10^–3^ −0.240	7.7 × 10^–3^	5.0	Pharmaceutical	100.30	^11^
RP-HPLC	10–60	1.220	5.0	Pharmaceutical	100.12	^12^
RP-HPLC	10–60	1.450	-	Pharmaceutical dosage form	99.95	^6^
HPLC	0.182–5.035	0.006	5.0	Rat plasma	94.80	^13^
HPLC-DAD	0.1–1.25	0.020	7.0	Human serum	98.44	^14^
				Pharmaceutical	100.10	
Electrochemical determination GCE	1.009–50.449	0.139 (DPV)	4.7 and 8.0	Human serum	99.62–106.23	^15^
BDDE	0.505–50.449	0.026 (DPV)		Pharmaceutical dosage form	99.96–100.71	
Electrochemical determination ACR@MIP/GCE	5.045 × 10^–6^- 5.045 × 10^–5^	1.756 × 10^–7^ (DPV)	-	Human serum	99.93 (DPV)	^16^
		1.428 × 10^–6^ (EIS)			97.50 (EIS)	
Electrochemical determination				Pharmaceutical	98.50–101.50	This work
Calix[4]arene/PPy/PGE Calix[6]arene/PPy/PGE Calix[8]arene/PPy/PGE	0.100–0.800 0.100–0.800 0.100–0.600	0.028 (DPV) 0.021 (DPV) 0.018 (DPV)	4.0	Urine	98.0–101.50	

This study presents the first voltammetric determination of tofacitinib citrate using a PGE modified with a novel calix­[n]­arene, addressing a previously unreported approach in the literature and demonstrating the originality and applicability of the proposed method.

Table 6 summarizes the comparative performance of the developed Calix­[n]­arene/PPy/PGE sensors against various reported analytical methods for Tofacitinib determination. The proposed electrochemical platform, particularly the Calix[8]­arene/PPy/PGE, demonstrates a competitive LOD of 0.018 ppm, which is superior to several chromatographic techniques and electrochemical sensors reported in the literature.

While molecularly imprinted polymer (MIP)-based sensors^16^ offer lower detection limits, our method provides a significant advantage in terms of simplicity and cost-effectiveness by utilizing disposable PGEs. The repeatability of the developed calix­[n]­arene/PPy/PGE sensor was evaluated by performing three consecutive measurements with the same modified electrode. The relative standard deviation (RSD) values were found to be between 0.317% and 1.298%, indicating excellent signal stability and precision during the analytical process. It is important to note that the proposed sensor is designed as a disposable (single-use) platform. Unlike conventional solid electrodes (e.g., GCE or Au) that require rigorous mechanical polishing and electrochemical activation between each analysis, the PGE offers a cost-effective and practical alternative. Utilizing a freshly modified PGE for each measurement inherently eliminates common issues such as electrode fouling, surface passivation, and cross-contamination, which are often encountered in the analysis of complex biological matrices like human urine. Therefore, the disposable nature of the sensor is a strategic advantage for rapid, routine clinical applications and point-of-care testing. The observed trend in LOD values (decreasing from Calix[4] to Calix[8]) suggests that the larger macrocyclic cavity of Calix[8]­arene facilitates a more effective host–guest interaction with Tofacitinib, thereby enhancing the sensitivity. Furthermore, the high recovery rates (98.0–101.5%) obtained in complex matrices like human urine and pharmaceutical dosage forms confirm that the sensor is highly resistant to matrix effects and suitable for routine clinical and quality control applications.

In conclusion, a novel and cost-effective electrochemical sensing platform based on Calix­[n]­arene-modified polypyrrole pencil graphite electrodes was successfully developed for the determination of Tofacitinib. Among the tested macrocycles, the Calix[8]­arene/PPy/PGE sensor exhibited the highest sensitivity with a remarkably low LOD of 0.018 ppm, suggesting that the larger cavity size of the octamer provides the optimal steric fit for the tofacitinib molecule. The synergy between the high surface area of the PPy film and the specific molecular recognition capability of the calixarene framework enabled superior analytical performance. The practical applicability of the sensor was validated in pharmaceutical dosage forms and human urine samples, yielding excellent recovery rates (98.0–101.5%). These findings suggest that the developed disposable sensor provides a powerful, fast, and inexpensive alternative to traditional chromatographic methods for routine clinical and pharmaceutical analysis.

## Data Availability

The data presented in this article are available within the manuscript.
